# Natural Course of Disease

**Published:** 1855-11

**Authors:** 


Natural Course of Disease.—Dr. Tholozan observes that the necssities of war furnish us with the opportunity of studying disease when left to Nature. If the surgeon pay to this study a sufficient attention, science and art will be gainers; for it will often occur to him to see diseases that are supposed to require the most active treatment, cured under the most unfavorable circumstances by the sole efforts of Nature. This arises from even the severest affections having their slight causes; and if, on the other hand, we may observe in the soldier disease in its most intense form, the multiplication of cases of the same kind, on the other hand, allows us to observe the diseases of armies in every shade of intensity.—(*Gazette Médicale.* No. 23.) *Ibid.*

